# Improvement of uncorrected visual acuity and contrast sensitivity with perceptual learning and transcranial random noise stimulation in individuals with mild myopia

**DOI:** 10.3389/fpsyg.2014.01234

**Published:** 2014-10-29

**Authors:** Rebecca Camilleri, Andrea Pavan, Filippo Ghin, Luca Battaglini, Gianluca Campana

**Affiliations:** ^1^Department of General Psychology, University of Padova, Padova, Italy; ^2^School of Psychology, University of Lincoln, Lincoln, UK; ^3^Human Inspired Technologies Research Center, University of Padova, Padova, Italy

**Keywords:** visual acuity, contrast sensitivity, myopia, perceptual learning, tRNS

## Abstract

Perceptual learning has been shown to produce an improvement of visual acuity (VA) and contrast sensitivity (CS) both in subjects with amblyopia and refractive defects such as myopia or presbyopia. Transcranial random noise stimulation (tRNS) has proven to be efficacious in accelerating neural plasticity and boosting perceptual learning in healthy participants. In this study, we investigated whether a short behavioral training regime using a contrast detection task combined with online tRNS was as effective in improving visual functions in participants with mild myopia compared to a 2-month behavioral training regime without tRNS (Camilleri et al., 2014). After 2 weeks of perceptual training in combination with tRNS, participants showed an improvement of 0.15 LogMAR in uncorrected VA (UCVA) that was comparable with that obtained after 8 weeks of training with no tRNS, and an improvement in uncorrected CS (UCCS) at various spatial frequencies (whereas no UCCS improvement was seen after 8 weeks of training with no tRNS). On the other hand, a control group that trained for 2 weeks without stimulation did not show any significant UCVA or UCCS improvement. These results suggest that the combination of behavioral and neuromodulatory techniques can be fast and efficacious in improving sight in individuals with mild myopia.

## INTRODUCTION

Perceptual learning has been found useful in improving visual abilities such as visual acuity (VA) and contrast sensitivity (CS), both in participants with deficits in visual cortical processing such as amblyopia (Polat et al., 2004; Levi and Li, 2009; Astle et al., 2011) and in participants with optical defects such as myopia or presbyopia (Tan and Fong, 2008; Polat, 2009; Polat et al., 2012). The mechanisms subtending such improvements involve neural plasticity. The link between neural plasticity and visual improvements is better defined in the context of amblyopia and is thought to be due to abnormal interactions between neurons tuned to specific orientations and spatial frequencies (Polat et al., 1997) and inter-ocular suppression at early cortical levels (Li et al., 2011). Mechanisms of improvement in participants with non-corrected refractive defects on the other hand is more puzzling and has been ascribed to an increase in neuronal signal-to-noise ratio able to buffer the blurred (noisy) images due to optical defocus (Tan and Fong, 2008). Improvements of uncorrected VA (UCVA) following perceptual learning in participants with mild myopia ranges from 0.16 (Camilleri et al., 2014) to 2.2 LogMAR (Durrie and McMinn, 2007), whereas improvements in uncorrected CS (UCCS) ranges from no improvements (Camilleri et al., 2014) to an improvement of 2.6 times respect to baseline UCCS at high spatial frequencies (Tan and Fong, 2008). These variations in improvements may be attributed to the different training procedures used in different studies (e.g., simple contrast detection vs. contrast detection under lateral masking conditions).

Although many studies support the view that a high degree of specificity of perceptual learning for simple stimulus attributes (Fiorentini and Berardi, 1981; Karni and Sagi, 1991; Poggio et al., 1992; Schoups et al., 1995; Campana and Casco, 2003; see Sagi, 2011 for a review) points to plasticity at early cortical sites (Schoups et al., 2001; Pourtois et al., 2008; Hua et al., 2010; Sale et al., 2011), more recent studies have demonstrated that, under appropriate conditions, perceptual learning is generalizable to other stimulus characteristics and other visual tasks altogether, suggesting that plasticity could also involve changes in the read-out of sensory neurons by higher-level neurons, or be distributed across multiple levels of the visual cortical hierarchy (Liu and Weinshall, 2000; Ahissar and Hochstein, 2004; Zhou et al., 2006; Webb et al., 2007; Xiao et al., 2008; Jeter et al., 2009; McGovern et al., 2012; Kumano and Uka, 2013). Despite this, the levels of processing where plasticity takes place when learning transfers to different tasks such as VA and CS continues to be a matter of debate (Zhai et al., 2013; Bonaccorsi et al., 2014; Zhang et al., 2014).

Neuromodulation techniques such as transcranial magnetic stimulation (TMS) or transcranial electrical stimulation (tES) have also been tested for the restoration of visual functions in people with abnormal cortical processing and are also considered useful in the understanding of visual functions at the cortical level. While TMS has been found useful in increasing CS up to 1 Log CS on medium–high frequencies, both temporarily (Thompson et al., 2008) and for extended periods (Clavagnier et al., 2013), tES (e.g., transcranial direct current stimulation, tDCS) has only shown a transient improvement of CS (Spiegel et al., 2013).

Online transcranial random noise stimulation (tRNS, a type of tES using alternating current with random frequencies and delivered during task execution) has recently been proven to be the most efficacious type of electrical stimulation for boosting perceptual learning in healthy participants (Fertonani et al., 2011; Pirulli et al., 2013).

To date, no techniques of neuromodulation have been used in an attempt to improve visual functions in participants with optical defects such as myopia. Thus, in the present study a short perceptual training regime in a contrast detection task using a single Gabor patch joined with tRNS was administered in order to investigate the effects of this combined approach on UCVA and UCCS in participants with mild myopia.

## MATERIALS AND METHODS

### PARTICIPANTS

Sixteen participants with mild myopia were recruited from the University of Padova (mean age of 24.12, ranging between 19 and 27). The first group of eight participants carried out a 2-week (eight sessions) behavioral training using a contrast detection task combined with online high-frequency tRNS (hf-tRNS). The second group of eight participants (control group) underwent the same training protocol but without tRNS. This was done in order to compare the effect of the combination of behavioral training + tRNS with the effect of the behavioral training alone (without tRNS) on UCVA and UCCS.

The participants fit the following inclusion criteria: refractive error up to –2 diopters (D) in either eye (minimum was –0.75 D), with astigmatism not exceeding –0.5 D in either eye. All tests as well as the training were administered binocularly and with no optical corrections. The participants had a stable refractive index for the 6 months prior to training. Exclusion criteria included any other ocular condition or cause for reduced VA other than simple myopia and/or mild astigmatism; these include diabetes mellitus, pregnancy, presence of myopia-related ocular complications and any previous ocular surgery. To ensure the inclusion and exclusion criteria, prior to training the participants carried out a detailed assessment by an optometrist. This assessment was repeated at the end of the training. Additionally, each participant in the tRNS group also filled in a questionnaire ensuring that all were eligible to undergo non-invasive brain stimulation. Any participant with a history of seizures, internal metal objects, or previous traumatic brain injury was excluded from our study. This study was approved by the local ethics committee.

### EXPERIMENTAL PROCEDURE

Before (pre-tests) and after the training (with tRNS; post-tests), UCVA and UCCS were assessed for each participant by using Landolt C and Grating tests of the Freiburg Visual Acuity Test (FrACT; Bach, 1996). After 3 months from the post-test, in a follow-up session, UCVA was tested again in order to see whether any UCVA improvement was maintained over time.

Stimuli (UCCS assessment) consisted of sinusoidal gratings presented in a circular window with a narrow Gaussian taper. Size of the gratings was 3^°^. Grating orientations used were 0^°^, 45^°^, 90^°^, or 135^°^. The task of the participant entailed discriminating the orientation of the grating at different spatial frequencies, ranging from 1 to 15 cpd, in separate blocks.

The Landolt C test was used to assess UCVA. The task of the participants was to indicate, in every trial, the orientation of the gap of the Landolt C out of eight possible orientations.

For both FrACT tests, the Best-Pest adaptive procedure was used to calculate the absolute threshold for each of these tests. Stimulus duration lasted until the participants’ response. An auditory cue was presented upon stimulus presentation and a different auditory cue was implemented as feedback for error responses.

The following behavioral paradigm described was used in an earlier study by Camilleri et al. (2014) in a 2-month perceptual training regime in individuals with mild myopia. It consisted of a two-interval forced choice (2IFC) task where the participants had to detect the presence of a single Gabor Patch, which changed in contrast according to the performance of the participant. The threshold corresponding to 79% of correct detection was determined by using a 1up/3down staircase procedure (Levitt, 1971). Stimuli used in the training comprised Gabor patches consisting of a cosinusoidal carrier enveloped by a stationary Gaussian. Standard deviation of the luminance Gaussian envelope (σ) was equal to the sinusoidal wavelength (λ); that is, the size of the Gabor patch covaried with its spatial frequency. Additionally, the spatial phase of the cosinusoidal carrier equalled to 0 (evenly symmetric Gabor patch). Stimulus duration lasted 200 ms. Participants underwent eight training sessions over 2 weeks (four consecutive sessions each week) and trained on four different orientations of the stimulus with a single spatial frequency, chosen according to the individual’s cut-off performance in the pre-test UCCS measurement, defined as the spatial frequency at which the estimated contrast threshold from pre-training UCCS measurements was 0.50 (Michelson contrast; Zhou et al., 2006). Since interleaving different stimulus conditions (roving) has been shown to hinder perceptual learning (Kuai et al., 2005; Herzog et al., 2012), in order to increase the efficacy of perceptual learning, participants were trained on the same orientation for two consecutive days. Three participants were trained with a spatial frequency of 11 cpd, two participants with 7 cpd, and the remaining three participants with a spatial frequency of, respectively, 5, 9, and 15 cpd. Each session consisted of eight blocks each containing 60 trials, which lasted for approximately 45 min. Participants were administered hf-tRNS (1.5 mA) during the first five blocks on each session (Fertonani et al., 2011). In order to reduce spatial and temporal uncertainty both an auditory and a spatial cue were implemented. On each trial a central fixation point preceded the presentation of each interval, and an auditory cue indicated when the stimulus (if present) appeared. Performance feedback was also provided to the participants in the form of an auditory beep following an incorrect response.

### APPARATUS

Stimuli were displayed on a 22-in Philips Brilliance 202P4 monitor with a refresh rate of 60 Hz. The monitor was luminance-calibrated with gamma = 1 by means of a professional monitor calibrator (Datacolor Spyder 4 Elite). The stimuli used in the training were generated with the Matlab Psychtoolbox (Brainard, 1997; Pelli, 1997), whereas stimuli for measuring UCVA and UCCS were generated using the Freiburg Acuity and Contrast Test (FrACT 3.8, Bach, 1996). Spatial dithering (Bach, 1997) and color bit stealing (Tyler, 1997) for increasing the depth of contrast resolution (12 bit) were enabled on the FrACT, thus allowing precise CS measurement. All stimuli were presented centrally. The screen resolution was 1280 × 1024 pixels, each pixel subtended 0.33 arcmin at a viewing distance of 3 m, and 0.67 arcmin at a viewing distance of 1.5 m. Viewing distance was equal to 3 m for pre- and post-tests, whereas the training was administered from 1.5 m (Durrie and McMinn, 2007; Tan and Fong, 2008). Both the tests and training were carried out in a dark, silent room. Background screen luminance (corresponding to mean luminance of Gabor stimuli) was 31.5 cd/m^2^.

### tRNS

High-frequency tRNS was delivered using a battery-driven stimulator (BrainSTIM, EMS) through a pair of saline-soaked sponge electrodes. The tRNS consisted of an alternating current of 1.5 mA intensity with a 0-mA offset applied at random frequencies. The frequencies ranged from 100 to 640 Hz.

The stimulations were applied for approximately 5 min during each of the first five training blocks (Fertonani et al., 2011). The total duration of stimulation was ∼25 min. The active electrode had an area of 16 cm^2^ and was placed over the occipital cortex measured at ∼3 cm above the inion. The reference electrode had an area of 60 cm^2^ and was placed extracephalically on the upper right arm. The current density was maintained well below the safety limits (always below 1 A/m^2^; Poreisz et al., 2007). The electrodes were kept in place with bandages.

## RESULTS

**FIGURE 1:**
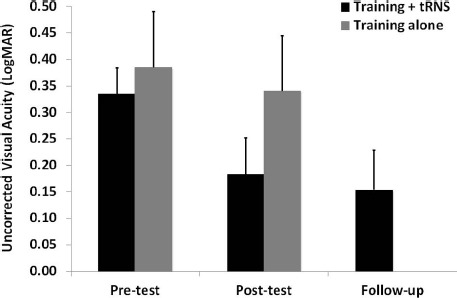
**Mean UCVA before (pre-test), after (post-test), and at 3 months follow-up (follow-up) of an eight-session contrast detection training, either coupled with online tRNS (black columns) or alone (gray columns).** Error bars represent one SEM.

**FIGURE 2:**
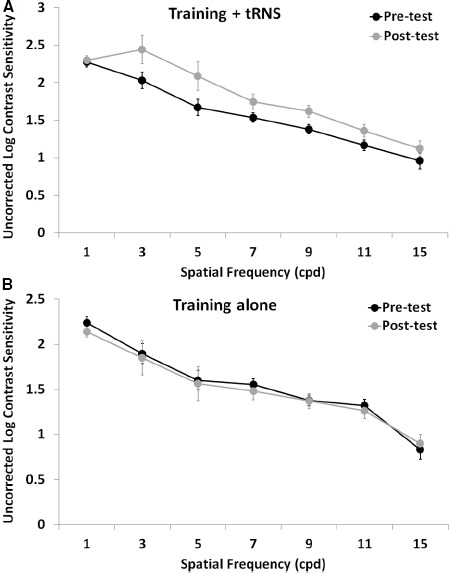
**Mean UCCS as a function of spatial frequency before (pre-test, black symbols) and after (post-test, gray symbols) an eight-session contrast detection training, either coupled with online tRNS (A) or alone (B)**. Error bars represent one SEM.

Pre-test, post-test, and follow-up measurements of the training + tRNS group were compared with a Friedman’s ANOVA, followed by two Bonferroni-corrected Wilcoxon signed-rank test. Friedman’s ANOVA revealed a significant UCVA difference between pre-test, post-test, and follow-up measurements ($\chi_{2}^{2}$ = 10.57, *p* < 0.005): participants trained on a contrast detection task for eight sessions with concurrent tRNS significantly improved their UCVA by 0.15 LogMAR (*Z* = –2.521, *p* < 0.05), that is from 0.33 to 0.18 LogMAR, and this improvement was maintained at the follow-up, where VA (0.15 LogMAR) was still significantly different from pre-test (*Z* = –2.37, *p* < 0.05; Figure 1, black columns). Across participants, the size of improvement ranged from virtually no change (only one participant, with an improvement <0.05 LogMAR; Camilleri et al., 2014) to an improvement of 0.33 LogMAR. UCCS (Figure 2A) also improved significantly at the following spatial frequencies: 3 cpd (*Z* = –2.521, *p* < 0.01), 5 cpd (*Z* = –2.38, *p* < 0.05), 7 cpd (*Z* = –2.24, *p* < 0.05), 9 cpd (*Z* = –2.521, *p* < 0.01), and 11 cpd (*Z* = –2.521, *p* < 0.01). The largest improvements were seen at intermediate spatial frequencies (3 and 5 cpd), where UCCS increased, on average, by five to six times at the post-test, with respect to the pre-test. Given that participants were mainly trained at higher spatial frequencies (just one participant was trained with 5 cpd, and none with 3 cpd), this means that learning mainly generalized from higher to lower spatial frequencies, consistently with previous studies (Astle et al., 2010). No significant improvement was seen at the lowest (1 cpd) and highest (15 cpd) tested spatial frequencies.

The control group (training alone) did not improve neither in UCVA (pre-test: 0.38 LogMAR; post-test: 0.34 LogMAR; *Z* = –0.098, *p* > 0.05; Figure 1, gray columns), nor in UCCS, in any of the tested spatial frequencies (all *Z* ≤ 1.4, *p* > 0.05; Figure 2B). Across participants and across spatial frequencies there was no substantial change in performance in post- with respect to pre-tests, except for two subjects who had a twofold increase in UCCS at 3 cpd.

## DISCUSSION

In a recent study, Camilleri et al. (2014) found that 24 sessions of contrast detection training in mild myopic participants produced a UCVA improvement of 0.16 LogMAR, but no UCCS improvement. In the present study, by using just eight sessions of training with a similar procedure but with the concurrent administration of tRNS, we found a comparable UCVA improvement (0.15 LogMAR), as well as a conspicuous improvement of UCCS at intermediate and high spatial frequencies (3–11 cpd). On the other hand, the same eight sessions of training with no tRNS did not produce any change in UCVA nor any substantial change in UCCS in any of the tested subjects. These results suggest that the application of tRNS during a perceptual training is able to boost perceptual learning (Fertonani et al., 2011; Pirulli et al., 2013), that is then transferred to other visual functions such as UCVA or UCCS at different spatial frequencies respect to the trained ones, under conditions of blurred vision due to optical defocus. The improvement in UCVA is smaller than that reported by other studies on myopia (Durrie and McMinn, 2007; Tan and Fong, 2008), and this is probably due to the use of a more efficacious training paradigm based on lateral masking (see Camilleri et al., 2014 for a discussion on this issue). However, the improvement in UCCS found in the present study (UCCS increased up to five to six times) is larger than that found in previous studies (up to 2.6 time of UCCS increase; Tan and Fong, 2008), and more pronounced at intermediate spatial frequencies, despite most participants were trained at higher spatial frequencies. These results, together with the striking difference in UCCS improvement respect to the study of Camilleri et al. (2014) where a similar training (yet longer and with no brain stimulation) was used, suggest that tRNS is particularly efficacious in boosting perceptual learning of UCCS, and its transferring of this learning to untrained spatial frequency channels.

The mechanisms mediating improvement of visual functions by perceptual learning in individuals with optical defects have not been completely understood. It has been suggested that refractive defects, often arising after the critical period, might produce a mismatch between an abnormal visual input due to optical defocus and a “normal” neural processing and connectivity developed (with focused input) in early childhood. Such a mismatch would decrease the perceived contrast, especially at high spatial frequencies, thus degrading UCVA (Tan and Fong, 2008). Learning to detect low contrast stimuli with no optical correction increase CS, thus improving the efficiency of neuronal responses to the abnormal (defocused) visual input, that in turn increases UCVA. However, learning of contrast detection requires a high number of training sessions (e.g., compare the results of Camilleri et al., 2014 with the present results). tRNS over the visual cortices could boost learning of (low) contrast detection. Being a repetitive and random sub-threshold stimulation, tRNS could induce temporal summation of small depolarizing currents that could interact with the ongoing activity of cortical neurons tuned to specific orientations and spatial frequencies and engaged in a contrast detection task, thus enhancing performance and inducing synaptic potentiation (Fertonani et al., 2011).

Since eight sessions of perceptual training with no tRNS does not seem to induce any improvement, it could be argued that perceptual learning here is not playing a role at all, and that the observed improvements are solely due to tRNS. In fact, previous studies found that anodal tDCS over the visual cortex can improve CS even in the absence of perceptual training (Kraft et al., 2010; Spiegel et al., 2013). It must be pointed out that the effects of tDCS and tRNS seem to be mediated by different neural mechanisms (Terney et al., 2008): while tRNS seems to act by increasing the activity of ion (sodium) channels and therefore by a temporal summation of small membrane potentials induced by consecutive openings of these channels (Terney et al., 2008), tDCS directly modulates the transmembrane potential (thus the firing rate) of individual neurons with a continuous flux of current that produces an initial facilitation often followed by adaptation to rebalance the modulation of ion channel conductance (Bindman et al., 1964; Fertonani et al., 2011), and that in turn could produce unpredictable results. In fact the effects of anodal tDCS on perceptual learning are conflicting: when administered over the visual cortex before a perceptual task (offline), it produced an improvement of learning within the same day (Pirulli et al., 2013), but it blocked consolidation of learning on a subsequent day (Peters et al., 2013). On the other hand, Pirulli et al. (2013) showed that perceptual learning in a visual discrimination task was increased only when hf-tRNS was administered concurrently (online) with the task, while no improvement was seen when it was administered alone (offline, with no concurrent task). This finding makes it very unlikely that the improvements in UCVA and UCCS were due to tRNS alone without any effect of the concurrent behavioral training.

Future studies are needed to assess whether more efficacious training protocols (e.g., those based on lateral masking) can also benefit of a concurrent tRNS for improving visual abilities in participants with cortical (such as amblyopia) or non-cortical visual deficits (e.g., refractive defects), and whether the improvement is long-lasting, as found with longer trainings without brain stimulation (Polat et al., 2004; Tan and Fong, 2008). Additionally, although tRNS does not result in any superficial skin sensations and thus participants are not directly aware that they are undergoing stimulation, making it unlikely that unspecific effects of stimulation occur, incorporating a sham tRNS group in subsequent studies is deemed necessary in order to account for any possible placebo effects. Although it has been demonstrated in healthy participants that hf-tRNS is more effective when administered during a perceptual learning task (Pirulli et al., 2013), it would also be worthwhile investigating whether hf-tRNS in the absence of any behavioral training could also bring about improvements in UCVA and UCCS in refractive defects.

In sum, these preliminary findings suggest that coupling a short contrast detection training with tRNS in participants with mild myopia results in an increased UCVA and UCCS, similar to or even larger than that seen with no tRNS and with a longer training using a similar training paradigm and stimuli parameters, but in the absence of brain stimulation.

### Conflict of Interest Statement

The authors declare that the research was conducted in the absence of any commercial or financial relationships that could be construed as a potential conflict of interest.
